# The incidence, root-causes, and outcomes of adverse events in surgical units: implication for potential prevention strategies

**DOI:** 10.1186/1754-9493-5-13

**Published:** 2011-05-20

**Authors:** Marieke Zegers, Martine C de Bruijne, Bertus de Keizer, Hanneke Merten, Peter P Groenewegen, Gerrit van der Wal, Cordula Wagner

**Affiliations:** 1 NIVEL, Netherlands Institute for Health Services Research, Utrecht, The Netherlands; 2 EMGO Institute, Department of Public and Occupational Health, VU University Medical Centre (VUmc), Amsterdam, The Netherlands; 3 Department of Sociology, Department of Human Geography, Utrecht University, The Netherlands; 4 Netherlands Health Care Inspectorate, Utrecht, The Netherlands

## Abstract

**Background:**

We need to know the scale and underlying causes of surgical adverse events (AEs) in order to improve the safety of care in surgical units. However, there is little recent data. Previous record review studies that reported on surgical AEs in detail are now more than ten years old. Since then surgical technology and quality assurance have changed rapidly. The objective of this study was to provide more recent data on the incidence, consequences, preventability, causes and potential strategies to prevent AEs among hospitalized patients in surgical units.

**Methods:**

A structured record review study of 7,926 patient records was carried out by trained nurses and medical specialist reviewers in 21 Dutch hospitals. The aim was to determine the presence of AEs during hospitalizations in 2004 and to consider how far they could be prevented. Of all AEs, the consequences, responsible medical specialty, causes and potential prevention strategies were identified. Surgical AEs were defined as AEs attributable to surgical treatment and care processes and were selected for analysis in detail.

**Results:**

Surgical AEs occurred in 3.6% of hospital admissions and represented 65% of all AEs. Forty-one percent of the surgical AEs was considered to be preventable. The consequences of surgical AEs were more severe than for other types of AEs, resulting in more permanent disability, extra treatment, prolonged hospital stay, unplanned readmissions and extra outpatient visits. Almost 40% of the surgical AEs were infections, 23% bleeding, and 22% injury by mechanical, physical or chemical cause. Human factors were involved in the causation of 65% of surgical AEs and were considered to be preventable through quality assurance and training.

**Conclusions:**

Surgical AEs occur more often than other types of AEs, are more often preventable and their consequences are more severe. Therefore, surgical AEs have a major impact on the burden of AEs during hospitalizations. These findings concur with the results from previous studies. However, evidence-based solutions to reduce surgical AEs are increasingly available. Interventions directed at human causes are recommended to improve the safety of surgical care. Examples are team training and the surgical safety checklist. In addition, specific strategies are needed to improve appropriate use of antibiotic prophylaxis and sustainable implementation of hygiene guidelines to reduce infections.

## Background

Adverse events are a major source of morbidity and mortality. Patient record review studies showed that the incidence of adverse events (AEs) varied from 3% to 17% among hospitalized patients [1-12]. Approximately 50% of the AEs were judged to be preventable. Most of the AEs resulted in minor or temporary disability, but a proportion of the AEs, 4% to 21%, contributed to death. All these studies have shown that a high percentage of AEs are attributable to surgical specialties, ranging from 51% to 77% [1,2,4,8-10,13,14]. Previous population-based record review studies that reported on AEs attributable to surgical specialties in detail are now more than ten years old [13,14]. Since then both surgical techniques and quality assurance have developed rapidly. Surgical techniques have been improved, increasing the opportunities for successful treatment of complex diseases. However, this also increases the potential for errors that can result in patient harm, leading to disability or death [15,16]. In the meantime, many solutions designed to reduce the risk associated with surgery have been developed.

Recent data are needed to provide insight into the scale, nature, causes and preventability of surgical AEs and to identify those prevention strategies which can reduce the main causes of surgical AEs. A large patient record review study on the occurrence of AEs was performed in order to assess the national incidence of AEs among Dutch hospitalized patients in 2004 [17,18]. We use the data from this study in our paper in order to present a detailed description of surgical AEs.

Our research questions were: (1) what is the incidence, consequences, nature, and preventability of surgical AEs among hospitalized patients, and (2) what are the causes of, and potential prevention strategies for, surgical AEs?

## Methods

### Study design and setting

We have performed a patient record review study in a random sample of 21 Dutch hospitals: 4 university, 6 tertiary teaching and 11 general hospitals. From each hospital we randomly selected 200 admissions (>24 hours stay) of discharged patients and 200 admissions of deceased hospital patients in 2004 - or less if the total number of patients who died in 2004 was lower. The study was carried out between August 2005 and October 2006. We selected admissions for 2004 in order to obtain a complete overview of the patient information, including a 1-year period after discharge or death of the patient [17].

In total, 3,983 admissions of deceased hospital patients and 3,943 admissions of discharged patients (>24 hours stay) were reviewed, excluding admissions in psychiatry, obstetrics and for children younger than one year-old. A large sample of deceased patients was included to assess more precisely the occurrence of potentially preventable deaths in hospitals. All figures in this paper were corrected for the sampling frame and were representative of the Dutch population of hospitalized patients [18]. The methods were based on previous AE studies in other countries and have been described in detail elsewhere [17]. The study protocol was approved by the Amsterdam VU University Medical Center Ethics Board.

### Reviewer training

The hospital records of the selected admissions were reviewed by a team of 66 nurses and 55 medical specialists. The eligibility criteria for medical specialists to act as a reviewer were: more than 10 years post graduate general clinical experience; a good reputation among colleagues; that they had been retired for no longer than 5 years; that they had experience or affinity with the analysis of incidents, complaints and errors in clinical care; and that they were available for at least one day per week.

An additional expert panel of 18 medical specialists from several sub specialties was recruited to offer expert advice about accepted clinical practice during the review process. These specialists were authorities within their specialization and were recruited by the scientific associations of medical specialists. The panel consisted of specialists from all medical disciplines involved in the study.

The nurses and medical specialists followed a one-day training course in small groups comprising a maximum of 12 participants led by one researcher and one experienced nurse or medical specialist, respectively. During the training, the study protocol, definitions, and electronic review forms were explained and examples of AEs were discussed. The reviewers practiced on examples of cases and with the review forms. They were provided with a review manual in which the research protocol, instruments, and definitions were defined [17]. After one month of reviewing, they undertook a half-day training session to discuss their problems concerning the review process. The reviewers were also updated with the latest insights about the review process. These training sessions were repeated frequently during the data collection period. The problems discussed were collected and noted in a regularly updated Frequently Asked Questions (FAQ) document, which was distributed to all reviewers.

### Structured review of patient records

The nursing, medical and, if available, outpatient records of the 7,926 sampled hospital admissions were reviewed in a three stage review process. In the first stage, the nurse reviewers screened the records by using 18 explicit screening criteria indicating potential AEs (Appendix A).

In the second stage of the review process, two medical specialists independently reviewed the records screened positive by the nurses in stage one (4,317 patient records). During the previous stage, nurses indicated which medical specialty would be most suitable for reviewing each particular record. Two physician reviewers from the specialty indicated reviewed each record independently and determined whether an AE had occurred.

Records with screening criteria concerning surgical care were reviewed by two surgeons. They used a standardized procedure with a structured electronic review form to determine whether an AE had occurred and to what degree it was preventable. The determination of an AE was based on three criteria: (1) an unintended physical and/or mental injury; which (2) resulted in temporary or permanent disability, death or prolongation of hospital stay, and; was (3) caused by health care management rather than the patient's disease (Appendix A) [1-3,8,17]. *Preventability *was defined as care that fell below the current professional standards and expected performance for practitioners or systems. Preventability was measured on a six-point scale (one = no preventability and six = definite preventability). Consistent with most previous international studies, we used a score of one to three to indicate that AEs were not preventable and a score of at least four to indicate that AEs were preventable [18].

If there was disagreement about the presence and/or preventability of an AE between the two independent surgeon reviewers, they started a procedure to achieve a consensus (stage 3). If the two surgeons could not reach a consensus, a third surgeon reviewer with access to all the information determined the final judgment.

If an AE was identified, the consequences, most responsible specialty, causes and potential prevention strategies were assessed. *Surgical AEs *were defined as AEs attributable to surgical treatment and care processes. The surgeon reviewers classified the surgical AEs by clinical procedure involved, such as diagnostic process, surgical procedure, drug/fluid, medical procedure, other clinical management, discharge, and other. The c*onsequences *were defined as: a prolonged hospital stay, extra treatment, a readmission to the hospital, extra outpatient care, a temporary or permanent disability at discharge, and death as a result of an AE. The consequences of surgical AEs were also classified by the type of *injury*, such as: bleeding, infection, shock, thrombosis, necrosis, fistula forming, and abnormal wound healing. The classification by injury was according to the national reporting system of adverse outcomes developed by the Association of Surgeons in The Netherlands [19,20]. For each AE, the reviewers indicated all consequences.

In addition, the underlying *causes *of AEs were assessed. An AE arises often due to several causal factors, including technical, organisational, human, and patient-related factors. For each AE, the surgeon reviewers selected one or more causes. The reviewers selected all causes that contributed to the occurrence of the AE. They used a recognized taxonomy of root causes: the Eindhoven Classification Model of PRISMA-Medical, a root cause analysis tool [21,22]. The categories of the taxonomy are:

- Human factors which are, skill-based, for example failures in the performance of highly developed skills, or, rule-based, for example an incorrect fit between an individual's training or education and a particular task, or knowledge-based, for example inadequate application of existing knowledge;

- organizational factors, for example inadequate or unavailable protocols, management priorities, inadequate transfer of information and cultural aspects;

- technical factors, for example material defects, failures due to poor design of equipment, software, labels or forms;

- patient-related factors, for example, co-morbidity, age, and treatment compliance [23].

Finally, for all preventable AEs, *potential prevention strategies *were selected. The categories were based on PRISMA and the Canadian patient record review study [1,22]. The review form distinguished ten prevention strategies: quality assurance/peer review, training, evaluation, procedures, motivation, information and communication, technology/equipment, personnel, scaling up, and financial investment. For each AE, the surgeon reviewers could select one or more potential prevention strategy. The reviewers selected the causes and potential prevention strategies based on information in the patient record and on their perception of the situation [23].

### Statistical analysis

The incidence, preventability, nature, consequences, causes and potential prevention strategies of surgical AEs were analyzed using descriptive statistics and frequency tables. To convert the study sample into results representative of the Netherlands, a weighting factor was used to correct for over-representation of patients admitted to a university hospital and patients who died in hospital in the study sample. The weighting factor was the inverse of the probability of being included in the sample and was calculated by dividing the representation of a group in the population by the representation of this same group in the study sample [18]. All statistical analyses were performed using SPSS 14.0.

For all the comparisons of incidence rates, consequences and causes, tests for the comparison of proportions in two independent groups were used, corrected for binomial distribution [24].

## Results

### Incidence and preventability

In the 7,926 reviewed patient records, 744 AEs were found. Using the weighting factor for over-representation of deceased patients and hospital type, the results showed that surgical AEs occurred in 3.6% of hospital admissions and accounted for 64.5% of all AEs (Table 1). The incidence rate of surgical AEs is significantly higher than the incidence rate of non-surgical AEs (2.1%). Of all surgical AEs, 40.5% were judged to be preventable. Table 2 presents examples of surgical AEs identified in this study.

**Table 1:** Occurrence and preventability of surgical AEs compared with other AEs

AEs, % (95% CI)	Surgical AEs	Other AEs	Total
In total population^a^	3.6 (3.1-4.2)	2.1 (1.7-2.5)	5.7 (5.1-6.4)
Of all AEs^a^	64.5 (59.0-69.6)	35.5 (30.4-41.0)	100
Preventable^a^	40.5 (33.7-47.7)	38.4 (30.0-47.6)	39.6 (34.4-45.4)

^a ^Corrected for the over-representation of deceased patients and hospital type

**Table 2:** Case descriptions of surgical adverse events

***Non preventable surgical adverse events***

Pneumonia after thoracotomy, resulting in artificial ventilation and antibiotics
Adverse drug (propofol and sufentanil) reaction (bronchospasm and exanthem), resulting in extra treatment with medication
Incisional hernia after laparotomy resulting in readmission and reoperation
Infection tissue expander head, resulting in a readmission, operative removal and a reconstructive procedure
Wound leakage and sepsis after colorectal anastomosis, resulting in a reoperation, ICU admission (artificial ventilation), prolonged hospital stay and repeated outpatient clinical visits

***Preventable surgical adverse events***

Surgery taking place at the wrong site during kidney transplantectomy, resulting in an extra incision of the skin
Urosepsis after operation on femur fracture caused by non-indicated CAD
Technical inadequate hip prosthesis, resulting in two repositions and reoperation
Inadequate nasal intubation by tonsillectomy, resulting in tear off concha, bleeding and reoperation
Spinal anaesthesia in non-treated hypertension, resulting in hypotension, coma and contributed to death.

### Consequences

**Figure 1:**
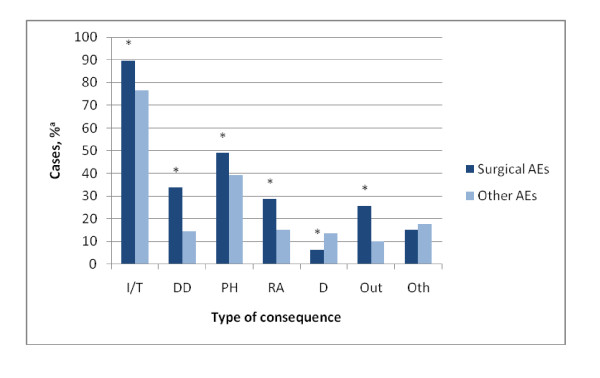
**Consequences of surgical AEs compared with other AEs**. I/T indicates intervention/treatment; DD, disability at discharge; PH, prolonged hospital stay; RA, readmission to the hospital; D, death; Out, extra outpatient care; and Oth, other. ^a ^Corrected for the over-representation of deceased patients and hospital type * Significant difference between surgical AEs and other AEs (P < 0.05)

Surgical AEs contributed less often to patients' deaths compared to other types of AEs (6.5% [5.3% -7.9%] vs. 13.6% [10.5% - 17.5%]; z = 2.47; P <.01) (Figure 1). The other consequences of surgical AEs were more severe compared to other types of AEs. Surgical AEs more often led to extra interventions or treatments (89.4% [95% CI, 84.6%-92.8%] vs. 76.7% [68.1%-83.5%]; *z *= 3.76; P <.01), disability at discharge (33.7% [27.2% - 40.9%] vs. 14.6% [8.9% - 23.1%]; z = 4.54; P <.01), readmissions to the hospital (28.8% [22.6% - 35.9%] vs. 14.7% [9.0% - 23.1%]; z = 3.32; P <.01), prolonged hospital stay (48.9% [41.9% - 56.1%] vs. 39.4% [30.8% - 48.8%]; z = 2.02; P <.05), and more outpatient visits (25.6% [19.7% - 32.4%] vs. 9.7% [5.2% - 17.5%]; z = 4.04; P <.01).

### Nature

More than half of the AEs attributable to anesthesiology, plastic surgery and orthopedics were judged to be preventable (Table 3). AEs attributable to vascular surgery (23.3%), anesthesiology (22.2%), heart/thorax surgery (19.2%), and plastic surgery (18.2%) contributed relatively often to permanent disability including death.

**Table 3:** Surgical AEs by specialty and proportions with preventability or permanent disability (including death)

Specialties	No. AEs	Preventable (Row %*)	Permanent disability (including death) (Row %*)
Anesthesiology	9	60.0	22.2
Plastic surgery	6	54.5	18.2
Orthopedics	41	52.2	8.6
Gynecology	12	50.0	14.3
General surgery	162	44.9	9.5
Ophthalmology	3	40.0	0
Dentistry/oral surgery	7	33.3	0
Heart/thorax surgery	41	32.0	19.2
Urology	21	30.8	3.8
Neurosurgery	14	26.7	0
Vascular surgery	41	25.0	23.3
Ear, nose and throat	10	14.3	0
Total	367	40.5	10.4

* Corrected for the over-representation of deceased patients and hospital type

The AEs attributable to surgical specialties were classified by clinical procedure (Table 4). Most of them were a result of surgical procedures (83.1%). Some surgical AEs were a result of medical procedures (5.5%), for instance urinary tract infection by bladder catheterization and lung bleeding after insertion of a Swann Ganz catheter. 3.7% of the surgical AEs were drug-related, such as administering the wrong type of medication, under- or overdoses of medication, or adverse drug reactions. Surgical AEs classed as 'other clinical management' (2.8%) were related to insufficient post operative care, for example inadequate administration of gastro-enteral feeding resulting in aspiration or a liver abscess drain becoming disconnected, resulting in sepsis. Examples of diagnosis-related surgical AEs (2.5%) were: 'missed intra-abdominal perforation' which resulted in a sepsis, 'missed incarcerated inguinal hernia', and 'missed appendicitis'. All surgical AEs related to 'other clinical management', the diagnostic process and discharge procedure were judged to be preventable.

**Table 4:** Surgical AEs by clinical procedure and proportions with preventability

Classification	No. (%*)	Preventability (%*)
Surgical procedures (Operative procedures)	292 (83.1)	34.7

Medical procedures (e.g. central catheters, endoscopies, pacemakers, intervention radiology)	19 (5.5)	55.6

Drug (e.g. side effects, allergic reactions, anaphylaxis)	13 (3.7)	50.0

Other clinical management (including nursing and allied health care)	15 (2.8)	100

Diagnostic process (e.g. missed, delayed or inappropriate diagnostic process)	22 (2.5)	100

Discharge procedure (e.g. inappropriate discharge)	2 (1.2)	100

Other (e.g. fall)	4 (1.2)	50

Total	367 (100)	40.5

* Corrected for the over-representation of deceased patients and hospital type

The most frequent injuries as a result of surgical AEs were: inflammation/infection (39.3%); bleeding/hematoma (23.1%); injury by mechanical/physical or chemical cause (22.1%); and other functional impairment (16.5%), like urine retention, respiratory insufficiency, renal insufficiency, anuria, strangulation of the ileus, and dystrophy of the hand after an operation for carpal tunnel syndrome (Table 5). AEs with a high percentage of preventability were pressure ulcers, injury by mechanical/physical or chemical cause, fistula forming, shock, and ischemia/heart failure. Injuries like shock and ischemia/heart failure contributed relatively often to permanent disability including death.

**Table 5:** Surgical AEs by injury (n = 596) and proportions with preventability or permanent disability (including death)

Injury	No. (Column %*)	Preventable (Row %*)	Permanent disability (including death) (Row %*)
Inflammation/infection	136 (39.3)	25.7	11.1

Bleeding/hematoma	72 (23.1)	25.8	6.3

Injury by mechanical/physical or chemical cause (e.g. puncture, perforation, joint or implant luxation)	46 (22.1)	68.3	6.1

Other functional disorder	49 (16.5)	35.6	15.8

Accumulation/leakage of body fluids	46 (12.2)	45.5	14.7

Abnormal wound healing (e.g. wound dehiscence/delayed fracture healing/pseudarthrosis/stenosis)	39 (12.0)	31.3	6.3

Symptoms without diagnosis (e.g. fever, pain)	7 (4.1)	16.7	0

Fistula forming	18 (4.0)	54.5	9.1

Shock	47 (3.9)	54.5	50.0

Necrosis/infarction	44 (3.8)	40.0	40.0

Thrombosis/Embolism	25 (3.5)	30.0	40.0

Ischemia/heart failure	40 (2.9)	50.0	42.9

Pressure ulcers	8 (2.9)	71.4	0

Rejection/allergy/other immunological reaction	2 (1.4)	0.0	0

Other/non-specified category	9 (3.7)	40.0	10.0

* Corrected for the over-representation of deceased patients and hospital type

### Causes and prevention strategies

**Figure 2:**
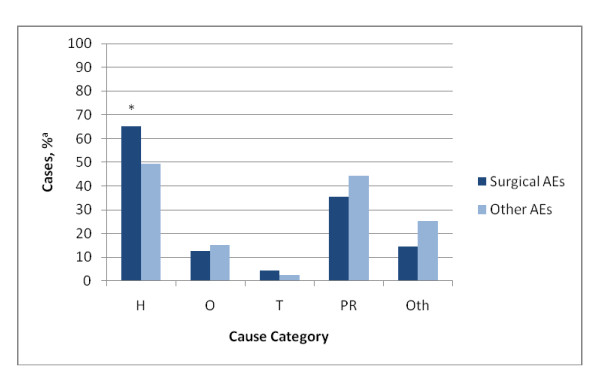
**Main categories of causes of surgical AEs compared with other AEs**. H indicates human; O, organization; T, technical; PR, patient related; and Oth, other. ^a ^Corrected for the over-representation of deceased patients and hospital type * Significant difference between surgical AEs and other AEs (P < 0.05)

The surgeon reviewers selected 653 causal factors for the 367 surgical AEs found in this study. Causal factors of surgical AEs were judged to be predominantly human (65.2%) and patient-related (35.3%), and less often organizational (12.7%), and technical (4.4%) (Figure 2). Causes for surgical AEs were significantly more often related to human factors than in the other types of AEs (65.2% [95% CI, 58.1% - 71.7%] vs. 49.9% [40.8% - 59.0%]; z = 3.25; P <.01). Causes related to patients were less often the causal factor of surgical AEs compared to other types of AEs (35.3% [28.8% - 42.4%] vs. 44.4% [35.6% - 53.6%]; z = 1.91; P = .06).

The surgeon reviewers recommended 442 potential prevention strategies for all 150 surgical AEs that were judged to be preventable (Table 6). For more than 70% of the surgical AEs, quality assurance/peer review was selected as a potential prevention strategy. Other frequently selected prevention strategies were training (58.9%), evaluation (51.4%), and improving procedures (40.6%).

**Table 6:** Recommended potential strategies (n = 442) to avoid preventable surgical AEs (n = 150)

Potential prevention strategy	No. preventable AEs	Frequency (%)*
Quality assurance/peer review (Continuously monitoring quality and assessment of health care workers performance by individuals in the same field)	111	72.9

Training (improving (re) training programs for skills needed)	78	58.9

Evaluation (evaluating the current way of behaving regarding safety)	84	51.4

Procedures (improving formal and informal procedures)	65	40.6

Motivation (positive behavior modification)	41	30.2

Information and communication (improving available sources of information, communication structures and medical record keeping)	33	17.3

Technology/equipment (e.g. redesign of technical devices)	11	9.3

Personnel (Increasing amount of personnel)	4	3.1

Scaling up (handling the problem at a higher organizational level)	4	1.8

Financial investment (Financial investments in required areas)	3	1.5

Other	8	3.6

* Corrected for the over-representation of deceased patients and hospital type

## Discussion

### General findings

In this study the incidence rate of surgical AEs is 3.6% of all hospital admissions. Of all in-hospital AEs, 65% were attributable to surgical specialties of which 41% were considered to be preventable. The scale of surgical AEs is comparable to findings in previous studies [1,2,4,8-10,13,14]. After years of improvements in patient safety [25] the scale of surgical AEs is still considerable. Surgical care is developing continuously. Over the past decades, the number and complexity of diagnostic procedures and therapeutic interventions have increased markedly. More diseases can be treated successfully and vulnerable patients are receiving surgical treatment more often. Surgical interventions by their nature are associated with a higher degree of risk. The increasingly complicated techniques and innovations, however, also enhance the potential for error [15,16,26].

Surgical AEs led more often to permanent disability, unplanned readmissions, unnecessary treatments and outpatient visits than other types of AEs. Our study showed that infection, bleeding and injury due to a mechanical/physical or chemical cause formed the largest group of injuries as a result of surgical AEs, reflecting the results of patient record review studies of more than ten years ago [13,14]. The high percentage of infections and bleeding raises the question whether evidence-based prophylactic protocols and guidelines have been successfully implemented [13,14]. More than two-thirds of the injuries related to a mechanical/physical or chemical cause was judged to be preventable. The surgeon reviewers noticed that an inaccurately performed procedure was a major contributing factor to injuries by puncture or perforation of other organs, such as nerves, vessels and bowels. Moreover, specific techniques that are not carried out according to the latest guidelines resulted in AEs such as leakage and wound dehiscence. Also the inadequate application of new techniques, such as endoscopic procedures, and procedures that demand specific expertise and experience, for example vessel surgery, resulted in AEs.

In this study, causal factors of surgical AEs were judged to be predominantly human factors (65%) and less often organizational (13%) or technical (4%). Given the high degree of preventability of surgical AEs that resulted in injury by mechanical/physical or chemical cause, the frequently recommended prevention strategies were: more training; improving procedures; and quality assurance.

### Strengths and limitations

A strong aspect of the study is the large number of patient records that were reviewed systematically. The use of a random, stratified, sample of diverse patient admissions and a systematic screening process led to results representative of Dutch hospitals. Also, AE criteria are well known, which allows comparisons with other studies. A patient record review of hospitalized patients is by far the most applied and thoroughly studied method for the assessment of the occurrence and nature of AEs [27]. The record review method has, however, some limitations. Because it depends on the completeness of the information that is recorded in the patient record, the amount of surgical AEs identified in this study could be underestimated. It is unknown whether the detection of AEs is equal for surgical and non-surgical AEs. If surgical AEs are easier to recognize in medical records, the difference between surgical and non-surgical AEs may be overestimated. Moreover, the inter-rater agreement for the judgment of AEs was fair for the assessment of AEs (κ = 0.25) and their preventability (κ = 0.40) [18].

Record review may not be the most accurate method to get an insight into the technical and organizational causal factors. Because technical and organizational factors are less often reported in the patient records, they may have been under-reported in this study [23]. An approach that combines a record review with prospective methods in which clinical staff are interviewed about the origin of the AEs will give a more appropriate and complete picture of the organizational and technical causes. The combination of record review and interviews with clinical staff may compensate for the lack of information in patient records [28].

The aim of the Dutch Adverse Event Study was to measure a national incidence rate for hospital AEs. An in-depth analysis of surgical AEs based on the national study is hampered by the small numbers of AEs for some sub categories. The results of the categories with a small number of AEs should be interpreted with caution.

### Implications for practice

Interventions to reduce surgical AEs based on evidence-based studies, systematic reviews and ongoing clinical trials are increasingly available. In Table 7, we present well-known interventions tailored to the main prevention strategies recommended in this study.

**Table 7:** A selection of well-known interventions to reduce surgical adverse events tailored to the selected prevention strategies in this study

*Main potential prevention strategies selected by the surgeon reviewers (see Table 6)*	*Interventions to reduce the incidence of surgical adverse events*
Quality assurance/peer review (Continuously monitoring quality and assessment of health care workers performance by individuals in the same field)	Patient record review [44,45] Morbidity and mortality conferences [46,47] Incident reporting [44,48]

Training (improving (re) training programs for skills needed)	Training for improvement of skills and for implementation of new techniques (e.g. simulation training) [29-31] Improving training and supervision of residents [26]

Evaluation (evaluating the current way of behaving regarding safety)	Multisource feedback to asses performance [49] Portfolio of competence and performance [50]

Procedures (improving formal and informal procedures)	Localizing specific surgical procedures and surgeries to high-volume centers [32,33,38] Improve compliance to existing protocols and guidelines for infection prevention: appropriate use of antibiotic prophylaxis, hand hygiene, sterilization of instruments, minimum number of persons in the operation theatre, reduction of door movements during operations [15,38-43]

Information and communication (improving available sources of information, communication structures and medical record keeping)	Operation room briefing with team communication checklist [34-36] Application of aviation-style crew resource management to improve teamwork [37] Improvement of completeness and adequacy of patient information and record keeping [53]

The interventions are focused both on technical and non-technical aspects of surgical care. For example, simulation training [29-31] and the concentration of certain procedures [32,33] to improve the technical performance on the one hand, and medical team training and checklists to improve the communication between team members in the operation theatre, on the other [34-37]. Specific procedures to reduce infections comprise both consistently applying antibiotic prophylaxis, and better adherence to existing protocols and guidelines in the operation theatre, which includes issues such as hand disinfection, the number of persons in the operation theatre, the number of door movements, and the sterilization of instruments) [15,38-43].

Quality assurance, i.e. monitoring of the performance of professionals by individuals in the same field, may take the form of morbidity and mortality conferences, a patient record review, reporting and learning from safety incidents, and evaluating the competence and performance of health care professionals with multisource feedback systems and portfolios [44-50].

Several interventions are incorporated into national safety campaigns, such as infection prevention, reporting and learning from safety incidents, medical team training and the surgical safety checklist [15,51,52].

### Recommendations for future research

Not all interventions we suggested are evidence-based or evaluated for surgical care. Future research should evaluate the cost-effectiveness of the interventions, especially for surgical specialties. Several interventions are currently being evaluated in Dutch hospitals, such as medical team training, the surgical safety checklist, multisource feedback, and incident reporting. Some of the interventions have been familiar for decades, like protocols for hand hygiene and appropriate use of antibiotic prophylaxis. More research should be done to explore the barriers and drivers for sustainable implementation of patient safety interventions in health care.

## Abbreviations

AE = Adverse event.

## Competing interests

The authors declare that they have no competing interests.

## Authors' contributions

MZ wrote the manuscript, prepared the protocol and instruments of the study, and collected and analyzed the data. MCB contributed to the manuscript, the design and conception of the study. GK reviewed patient records and has been involved in revising the article critically for important intellectual content. HM and PPG have been involved in revising the article critically for important intellectual content. GW contributed to the design and conception of the study and has been involved in revising the article critically for important intellectual content. CW is the general supervisor of the Dutch patient safety research group, contributed to the design and conception of the study, and contributed to the manuscript. All authors read and have approved the final manuscript.

## Appendix A Definitions and outcome measures

Description of **18 screening criteria **for potential adverse events [17]

1. Unplanned admission before index admission (admission reasons are related to the index admission)

2. Unplanned readmission after discharge from index admission

3. Hospital-incurred patient injury (Permanent or temporary injury obtained (acquired) during index admission)

4. Adverse drug reaction

5. Unplanned transfer from general care to (an) intensive care (unit)

6. Unplanned transfer to another acute care hospital (after unexpected deterioration of the patient)

7. Unplanned return to the operating room

8. Unplanned removal, injury or repair of organ during surgery

9. Hospital-acquired infection or sepsis

10. Other patient complication

11. Development of neurological deficit not present on admission

12. Unexpected death

13. Cardiac or respiratory arrest

14. Injury related to abortion or delivery

15. Inappropriate discharge to home

16. Dissatisfaction with care documented in the medical record

17. Documentation or correspondence indicating litigation

18. Any other undesirable outcome not covered above

The determination of an **adverse event **was based on three criteria [17]:

1. an unintended (physical and/or mental) *injury *which

2. results in temporary or permanent *disability*, death or prolongation of hospital stay, and is

3. *caused by health care management *rather than the patient's disease

To determine whether the injury was **caused **by health care management or the disease process a 6-point scale was used:

1. (Virtually) no evidence for management causation

2. Slight to modest evidence of management causation

3. Management causation not likely (less than 50/50, but 'close call')

4. Management causation more likely (more than 50/50, but 'close call')

5. Moderate to strong evidence of management causation

6. (Virtually) certain evidence of management causation

Causation scores of 4 to 6 were classified as adverse events.

The degree of **preventability **of the adverse events was measured on a 6-point scale:

1. (Virtually) no evidence for preventability

2. Slight to modest evidence of preventability

3. Preventability not quite likely (less than 50/50, but 'close call')

4. Preventability more than likely (more than 50/50, but 'close call')

5. Strong evidence of preventability

6. (Virtually) certain evidence of preventability

Adverse events with a preventability score of 4 to 6 were defined as preventable.
